# Characterization of Genetic Variability of Venezuelan Equine Encephalitis Viruses

**DOI:** 10.1371/journal.pone.0152604

**Published:** 2016-04-07

**Authors:** Shea N. Gardner, Kevin McLoughlin, Nicholas A. Be, Jonathan Allen, Scott C. Weaver, Naomi Forrester, Mathilde Guerbois, Crystal Jaing

**Affiliations:** 1 Computations, Lawrence Livermore National Laboratory, Livermore, California, United States of America; 2 Physical and Life Sciences, Lawrence Livermore National Laboratory, Livermore, California, United States of America; 3 Institute for Human Infections and Immunity and Departments of Microbiology & Immunology and Pathology, University of Texas, Medical Branch, Galveston, Texas, United States of America; CEA, FRANCE

## Abstract

Venezuelan equine encephalitis virus (VEEV) is a mosquito-borne alphavirus that has caused large outbreaks of severe illness in both horses and humans. New approaches are needed to rapidly infer the origin of a newly discovered VEEV strain, estimate its equine amplification and resultant epidemic potential, and predict human virulence phenotype. We performed whole genome single nucleotide polymorphism (SNP) analysis of all available VEE antigenic complex genomes, verified that a SNP-based phylogeny accurately captured the features of a phylogenetic tree based on multiple sequence alignment, and developed a high resolution genome-wide SNP microarray. We used the microarray to analyze a broad panel of VEEV isolates, found excellent concordance between array- and sequence-based SNP calls, genotyped unsequenced isolates, and placed them on a phylogeny with sequenced genomes. The microarray successfully genotyped VEEV directly from tissue samples of an infected mouse, bypassing the need for viral isolation, culture and genomic sequencing. Finally, we identified genomic variants associated with serotypes and host species, revealing a complex relationship between genotype and phenotype.

## Introduction

Venezuelan equine encephalitis (VEE) virus (VEEV) is a mosquito-borne alphavirus capable of causing large outbreaks of encephalitis in humans and horses. Major equine-amplified epidemics dating to the early 20^th^ century have affected hundreds-of-thousands of people and economically important equids. VEE complex viruses are endemic to South and Central America, Mexico, and Florida [1]. Although the case-fatality rate of VEEV is low in human infections (usually less than 1%), infection is typically highly debilitating and sometimes results in permanent neurological sequelae [2]. Moreover, because the disease primarily occurs in isolated rural areas and typical infections initially present with nonspecific flu-like symptoms, many cases involving spillover from enzootic cycles go undiagnosed or are mistaken for other febrile diseases such as dengue [3].

Enzootic VEE is also of concern due to its high burden of endemic human disease. For U.S. war fighters engaged in a conflict in Latin America, either direct exposure to the enzootic cycle in rural or suburban regions, as documented in Panama [2, 4], Colombia [5], and Mexico, or infections in urban settings [3, 6–10] could inflict direct casualties and severely compromise their ability to fight.

There are three major challenges related to VEE that we believe can be solved using new approaches: 1) rapidly estimating the origin of a newly discovered VEEV strain; 2) estimating its equine and/or human amplification, and thus epidemic potential; and 3) predicting the human virulence phenotype of a newly discovered VEEV strain. Phylogenetic relationships of a diverse collection of VEEV strains have proved useful for identification of the genetic features leading to epidemic spread to humans and livestock of this zoonotic pathogen [11, 12]. Here, we exploit high-throughput technologies to characterize a large panel of strains, including both virulent and avirulent strains; geographically diverse isolates from South America, Central America, Mexico, Florida and Texas; and isolates of multiple serotypes from diverse hosts, including human outbreak strains.

We performed whole genome SNP analysis of all available VEE antigenic complex genomes, verified that these SNPs accurately recapitulated the phylogeny from whole genome multiple sequence alignment (MSA), and developed a high-resolution genome-wide SNP microarray. We analyzed a diverse panel of 133 VEEV isolates on the microarray to validate array-based SNP calls with previously sequenced strains, and to characterize the SNPs in unsequenced isolates and place them on a phylogeny with sequenced genomes. We explored the relationship between genome variation and serotype, identified a number of variants non-randomly associated with these phenotypes, and examined the distribution of these variants across the VEEV genome.

## Methods

### Whole genome SNP analysis

We applied the kSNP software to find SNPs in the 144 VEE antigenic complex genomes available as of June, 2014 [13, 14]. kSNP is an alignment-free method based on examination of *k*-mers (oligos of length *k*) in the genome sequences. We define a SNP locus by a sequence context of length *k* centered on the polymorphic base, with (*k*-1)/2 conserved bases on either side. For this study, we performed SNP analysis with *k* = 13. Note that, under this definition of SNP loci, multiple loci (corresponding to different variations of the *k*-mer context) may overlap the same positions in a multiple sequence alignment; in this case, each of the multiple loci is only considered to be present in the genomes in which the (*k*-1) base context is conserved. This alignment-free SNP discovery is useful for viruses in which there may be highly divergent and poorly alignable regions among a large group of sequences, and where conserved regions only exist among small subgroups of sequences. The kSNP approach is free of the bias that otherwise results from the choice of a reference sequence, or from considering only a subset of regions of the genome that can be easily aligned, and can be implemented at scales to hundreds of genomes. We calculated SNP-based phylogenetic trees using parsimony, maximum likelihood (ML), or neighbor joining (NJ). For NJ, we used the number of SNP allele differences between pairs of target sequences as the distance metric. We mapped SNP alleles to branches of the trees using kSNP.

### Tree comparisons

SNPs from the E1, E2, E3, and capsid genes were extracted for separate analyses by identifying those SNPs that occurred within the specified gene regions (Table 1). We constructed a full genome MSA using the MUSCLE software [15], and built parsimony trees from the MSA, from all SNPs, and from SNPs in each gene. We compared the MSA and gene-based trees to the all-SNPs tree by treating all trees as unrooted and examining the splits of isolates into pairs of groups on either side of each internal branch in the tree. For each tree we used the Perl script CompareTree.pl [16] to calculate the fraction of splits shared between the MSA or gene-based tree and the all-SNPs tree; this serves as a metric of similarity between the tree topologies. We also used Dendroscope [17] to generate tanglegrams, which display pairs of trees side by side with lines interconnecting corresponding taxa. To minimize the numbers of crossing lines between trees without changing the tree topologies, we performed a series of equivalent branch rotations using the algorithm in [18] before generating tanglegrams. The pattern of crossing lines remaining provides a direct visualization of the differences in tree structures.

**Table 1:** Gene regions from which SNPs were extracted.

Gene	Coordinates on TC-83 genome
E1	10000–11327
E2	8563–9843
E3	8386–8574
Capsid	7562–8396
All SNPs	1–11446

### Microarray probe design

We designed microarray probes for every SNP locus. Our probe design strategy maximized sensitivity and specificity based on extensive prior lab testing on a Roche NimbleGen microarray platform, where we demonstrated 99.52% SNP allele call rates and 99.86% accuracy [19]. After testing seven alternative probe design strategies, we determined that maximum sensitivity and SNP discrimination accuracy resulted if the SNP base was at the 13^th^ position from the 5’ end of the probe (the end farthest from the array surface), probes were between 32 and 40 bases long, and lengths were chosen to equalize hybridization free energy (*ΔG*) to the extent possible within the allowable length range. We found that probes shorter than 32 bases had high false negative rates, and longer probes did not discriminate well between alleles. We found that *ΔG* was a better predictor of hybridization than the melting temperature *T*_*m*_. Probe candidates with hybridization free energy below *ΔG*_min_ = -43 kcal/mol were shortened until either their free energy exceeded *ΔG*_min_ or they reached the minimum 32 bases. Probes were designed around the SNP on both the plus and minus strands, for all observed SNP alleles, and all surrounding sequence variants.

Probes for the plus and minus strands were not the reverse complements of one another because the SNP does not lie at the center of the probe. We included probes for all observed alleles on each strand, yielding at least four probes per SNP locus for biallelic SNPs. In addition, we captured any sequence variation outside of the conserved k-mer SNP context in multiple alternative probes for each allele, so that some biallelic loci had more than 4 probes. Finally, we trimmed probes from the 3’ end to remove any N’s or other ambiguous bases, and omitted them altogether if doing so resulted in a probe shorter than 32 bases. When a probe was a subsequence of any other, only the shorter of the two was kept. SNP microarrays were fabricated using the 12-plex 135K Roche NimbleGen array format with 89% of the probes tiled in duplicate.

### Array hybridization to VEEV cDNA samples

The VEEV cDNA samples were fluorescently labeled and hybridized to VEEV SNP arrays as described previously [20]. Briefly, fluorescent labeling of samples was performed using the NimbleGen One-Color DNA Labeling Kit (Roche). One μg VEEV cDNA was added to Cy-3 labeled random primers, followed by isothermal amplification at 37°C using Klenow polymerase. Labeled DNA was purified via isopropanol precipitation and resuspended in water for microarray hybridization. DNA samples were prepared for hybridization using the NimbleGen Hybridization Kit LS (Roche). Three μg of labeled DNA was hybridized to each array, incubating for 40–45 hours at 42°C. Arrays were washed using the NimbleGen Wash Buffer Kit (Roche). The fluorescent signal on the array was scanned using a 2 μm Roche MS200 fluorescent scanner. Array feature intensities were generated using the NimbleScan software available from Roche NimbleGen.

### Selection of VEEV isolates for microarray experiments

Based on temporal and geographic range, outbreak associations and prior sequences generated at UTMB, we identified, propagated, and isolated RNA for microarray experiments from 134 of the most representative strains. To enable comparison of array- and sequencing-based genotyping methods, we included 81 isolates that had previously been sequenced in the set of strains tested on the array. Three of the previously sequenced isolates and one unsequenced isolate were run on duplicate arrays, for a total of 138 arrays. The serotype, passage history, year and location of collection, and host of each strain are listed in S1 Table.

To test the array’s ability to genotype viruses directly from tissue samples, six day-old CD-1 mice were infected with VEEV vaccine strain TC-83 [21] via the intracranial route. Each mouse was infected with 10^4^ PFU in a 20 μL volume. Three biological replicate mice were infected and sampled. Brains were harvested two days later and homogenized in a 1:10 w/v solution. The suspension was clarified by centrifugation and stabilized in Trizol (Life Technologies). RNA was extracted and purified using the Direct-zol RNA MiniPrep kit (Zymo Research, Irvine, CA) according to the manufacturer’s instructions. Whole cDNA was synthesized, fluorescently labeled, and hybridized to the SNP microarray as described above.

Data from microarray experiments is available at the Gene Expression Omnibus (GEO) repository under accession GSE79530.

### Allele calling from SNP microarray data and concordance calculations

We used our previously developed analysis software to call alleles at each locus for each sample analyzed on SNP microarrays. The software fits a linear model of strand and allele effects to the log intensity data from all probes for the locus, and calls the allele as the one with the largest coefficient in the fitted model. Separating the strand and allele effects is necessary in order to compensate for the differing hybridization efficiencies often seen between forward and reverse strand probes.

Because our definition of a SNP locus requires conservation of the 6 bases on either side of the polymorphic base, array probes for one locus may hybridize to genomes in which a similar locus context is present. That is, loci that are considered to be different in the sequence analysis, but have 13-mer contexts that are identical except at one or two positions, may be difficult to distinguish by microarray probes. Therefore, our current array analysis software does not attempt to determine whether a locus is present or absent, and instead makes an allele call for every locus.

For isolates that had genome sequences available, we computed the concordance rate between the allele calls from the array and the genome sequence, defined as the percentage of loci present in the genome for which the array calls agreed. We also computed the numbers of allele differences between each array sample and each genome, and determined whether the closest genome was in fact the genome sequence for that strain.

### Analysis of phylogenetic relationships and evolution of VEEV strains from SNP microarray data

We used the genotype data from genomic sequences to create maximum parsimony phylogenetic trees, using Parsimonator (https://github.com/stamatak/Parsimonator-1.0.2). We generated 100 trees using different random number seeds, and selected the most parsimonious (that is, the tree requiring the smallest total number of nucleotide substitutions) for downstream analyses.

### Phenotype/genotype associations

We identified variable positions in the MSA and used these loci as an initial set for building decision tree classifiers, using the recursive partitioning algorithm implemented in the R function “rpart” from the package “mvpart” [22, 23]. The “rpart” algorithm is described in detail in [24]; briefly, it selects a series of variables (SNP loci) and values (alleles) that split the viral strains into groups with homogeneous phenotypes. Each split of a group into smaller subgroups is chosen to minimize the Gini index, a measure of total subgroup inhomogeneity.

To ensure there were sufficient samples in the training and test sets for each phenotype to be predicted, we defined a “host type” for each sample by categorizing hosts as “large” (humans and equids) or “small” (rodents and mosquitos). For each phenotype (serotype and host type), we built multiple tree classifiers using a 10-fold cross-validation scheme, in which classifiers were trained with 90% of the isolates and tested with the remaining 10%. The amount of pruning in each decision tree classifier was determined by a complexity parameter; the “rpart” algorithm automatically determined an optimal complexity, defined as the smallest parameter value that yielded a cross-validation error rate within one standard deviation of the minimum error rate. We then built a final decision tree for each phenotype with the full set of genomes, using the optimal parameter to control the complexity of the tree. For each phenotype, we tested initial locus sets consisting of all polymorphic loci present in the TC-83 reference genome, as well as restricted sets containing only non-synonymous loci within the genes encoding structural proteins, or within the envelope glycoprotein genes.

For each classifier, we computed an overall accuracy, defined as the percentage of phenotype predictions that were correct. We also computed performance metrics for each specific phenotype, treating the decision tree as a binary classifier; e.g. for classifying isolates as serotype IAB vs any other serotype. For each specific phenotype, we counted the true positives (TP), true negatives (TN), false positives (FP) and false negatives (FN), and used them to compute the accuracy = (TP + TN) / (TP + FP + TN + FN), positive predictive value (PPV) = TP/(TP + FP), negative predictive value (NPV) = TN/(TN+FN), true positive rate TPR = TP/(TP+FN), and true negative rate TNR = TN/ (TN+FP).

We also ranked loci according to their strength of association with serotype or host type according to Fisher’s exact test, as implemented in the R “fisher.test” function. We corrected p-values for multiple comparisons using the Benjamini-Hochberg method.

## Results

### Whole VEEV genome SNP analysis

To identify single nucleotide variations among VEEV strains, we applied the kSNP software to 144 VEE antigenic complex genomes. We identified 7,926 SNP loci among these strains. The numbers of SNPs identified in structural protein encoding regions are summarized in Table 2. The annotations, 13-mer contexts and reference genome alignments for SNPs identified by whole genome analysis are listed in S2 Table.

**Table 2:** Numbers of SNPs identified in VEEV genomes, by gene region.

Gene	Number of SNPs
E1	1268
E2	1384
E3	262
Capsid	937

When we reran the kSNP analysis, using as an outgroup four strains of eastern equine encephalitis virus (EEEV, the closest relative of VEE complex alphaviruses), the total number of SNP loci increased to 9,486.

### Phylogenetic tree construction

**Fig 1:**
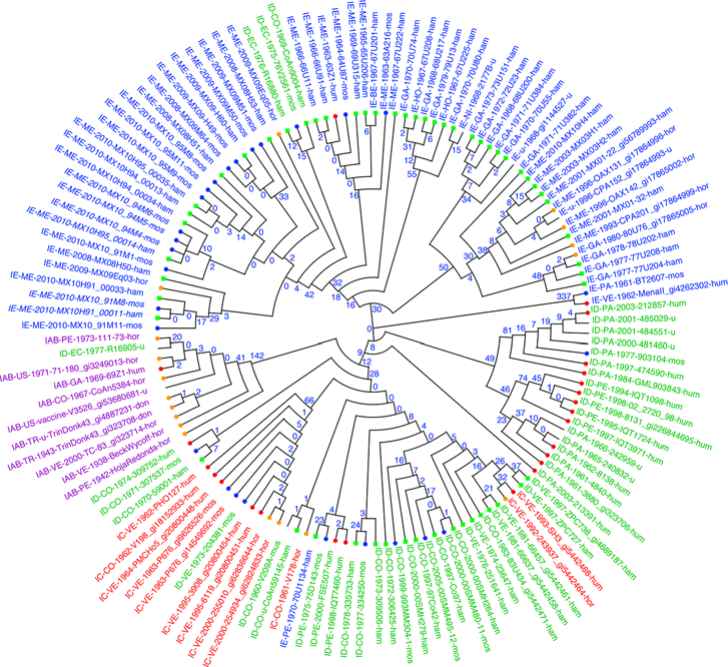
SNP phylogeny of VEEV isolates by parsimony. Strains are labeled by serotype-country-year collected-strain-host. Country codes are GA = Guatemala, PE = Peru, NI = Nicaragua, VE = Venezuela, CO = Colombia, TR = Trinidad, PA = Panama, US = USA, EC = Ecuador, ME = Mexico, BE = Belize, HO = Honduras, BR = Brazil, AR = Argentina, FG = French Guiana. Host codes are hor = horse, don = donkey, hum = human, mos = mosquito, ham = hamster, mus = mouse. u = unknown. Strains are colored by serotype (blue = IE, green = ID, red = IC, and purple = IAB). Hosts from which the strains were collected are indicated with symbols at the branch tips (red circles = human, orange circles = horses, blue circles = mosquitos, and green squares = hamsters). Counts of the number of alleles shared uniquely by the sequences down each branch are shown at the nodes in blue.

We then examined phylogenetic relationships among strains by building trees using different methods. First, we wished to determine which of the SNP-based tree construction methods performed best, by comparing the resulting trees to trees based on whole genome multiple sequence alignment (MSA). We found that the SNP tree built using maximum parsimony (Fig 1) was more similar to the MSA-based tree than those built with NJ or ML. Out of all splits in the alignment-based tree, 77% were also present in the parsimony tree, compared to only 68% in the ML tree. Moreover, the parsimony tree had fewer homoplastic SNPs than the ML tree (1679 versus 2153, respectively, from the dataset using the EEEV outgroup genomes). Homoplastic SNP loci are those in which the pattern of shared alleles does not conform to any of the branches of this tree, as a result of processes such as convergent evolution, homologous recombination, multiple mutations at the same site, or sequencing errors. Maximum parsimony has been shown to outperform ML in phylogenies that display heterotachy, a phenomenon in which the rates at which different nucleotide positions evolve change over time [25]. In this case, non-parametric estimation of trees by parsimony is more accurate than parametric methods such as ML.

Almost all VEEV strains could be uniquely identified by their genotypes according to variations across the identified SNP loci. Numbers at the interior nodes of the tree in Fig 1 indicate the number of loci at which a SNP allele is uniquely found in the descendants of the node and is shared by all of them. Only two sets of genomes were unresolved (i.e., had identical genotypes across all 7,926 SNPs); these strains are labeled in Fig 1 with italic type. One consisted of two genomes collected on successive days from Minatitlan, Mexico on August 26–27, 2010: MX10_91M8 from a mosquito pool and MX10H91_00011 from a sentinel hamster. The other comprised four genomes, also collected from Minatitlan in 2010; MX10_94M4, MX10_94M5 and MX10_94M6, collected from mosquito pools on August 26–27, and MX10H95_00014, collected from a hamster on August 28. These results confirm that sentinel hamsters do become infected with the variants circulating in mosquito vectors in the area at the same time. These isolates were members of a larger group of closely related genomes collected in Minatitlan, Mexico between July 2008 and late August 2010 from hamsters, mosquitos and two horses.

### Phylogenetic and phenotypic relationships of VEEV strains

To explore the relationship between the phylogenetic groupings of VEEV strains and their phenotypes, we examined the maximum parsimony tree shown in Fig 1, in which the genome annotations and plot symbols are color-coded by serotype and host, respectively. We observed a number of interesting patterns. First, we extended previous results [26] showing that VEEV strains with high overall similarity across the entire genome may exhibit different serotypes. For example, the epizootic serotype IAB strains and associated vaccine strain TC-83 (purple in Fig 1) collected from multiple countries from 1938–1973 form a distinct clade of highly similar isolates; however, this clade also included a serotype ID isolate (R16905) collected in 1977. In general, we saw that broad phylogenetic groupings were not exclusively associated with particular serotypes.

Similarly, we found that phylogenetic groupings were not strongly associated with particular hosts; the broad associations that did appear were likely artifacts of the different sampling strategies used for enzootic (serotype ID and IE) strains, which account for all samples from mosquitos and sentinel hamsters, and for epizootic (serotype IAB and IC) strains, which comprise most samples from equids and humans.

Finally, when we examined the collection dates of samples found in each major clade, we found that many clades were remarkably persistent. For example, the serotype IAB epizootic strains (and associated type ID outlier) showed little genetic variation, even though they were collected over nearly 40 years (1938–1977) across a wide geographic area, from the USA through Guatemala and Trinidad down to Venezuela and Peru, likely the result of incompletely inactivated vaccines made from older strains initiating later outbreaks [27]. Likewise, the serotype IC and ID isolates comprising the lower part of the tree in Fig 1, collected between 1961 and 2005, had very few differences across our panel of SNP loci.

### Association between genotypes and phenotypes

**Fig 2:**
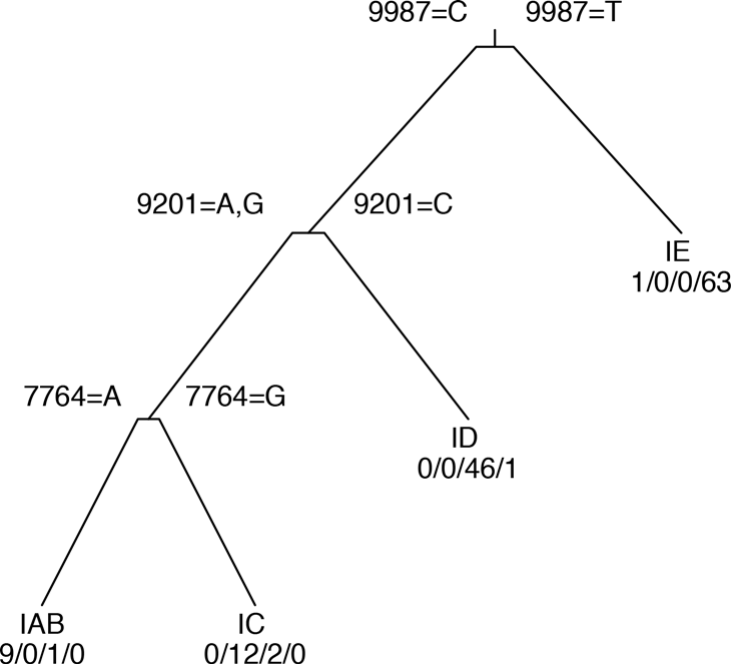
Decision tree for prediction of serotype from SNP alleles. Notations above internal nodes indicate SNP position in the TC-83 genome and alleles corresponding to left and right branches. Numbers below terminal nodes are numbers of isolates in node with serotypes IAB/IC/ID/IE respectively.

**Fig 3:**
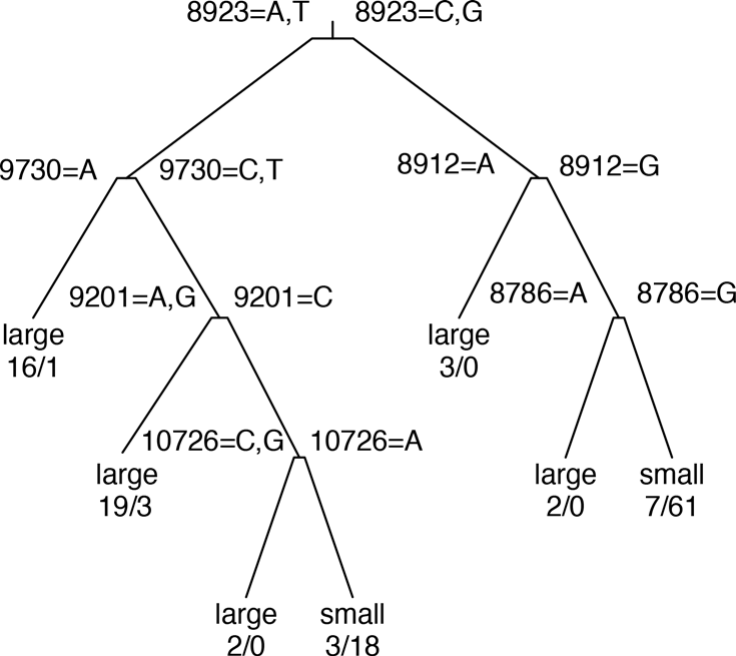
Decision tree for prediction of host type from SNP alleles. Notations above internal nodes are as in Fig 2. Numbers below terminal nodes are numbers of isolates in node collected from large/small host types, respectively.

Because the host and serotype associated with a VEEV isolate are not completely predictable from its position in the phylogeny, we searched for SNP loci that were associated with these important phenotypes for which the association was not simply a product of ancestry. We applied the “rpart” recursive partitioning algorithm to identify variations that are associated with particular host types or serotypes. The resulting decision tree classifiers are diagrammed in Figs 2 and 3. Our results indicate that these phenotypes are complex polygenic traits affected by multiple alleles on multiple genes. In each decision tree, the notations displayed above each branch point indicate the loci and alleles used in the associated test criteria; the annotations below each leaf node indicate the most common serotype or host type, together with the actual numbers of isolates at the leaf having each phenotype. For example, in the serotype tree (Fig 2), the first branch point separates the isolates according to the allele at alignment position 9987. Those with an T allele are classified as serotype IE; the remainder are then tested at position 9201, with a C allele indicating serotype ID; the rest are tested at position 7764, with an A allele indicating serotype IAB and a G indicating serotype IC.

Depending on the true serotype of the isolate, serotype prediction accuracy ranged from 95.6% for ID to 98.5% for IAB and IE strains (Table 3). Serotype IE was almost universally associated with a T allele at position 9987, which is in the p6K/TF gene.

**Table 3:** Accuracy, positive (PPV) and negative predictive value (NPV), true positive (TPR) and negative (TNR) rates for serotype and host type predictions.

Phenotype	Accuracy	PPV	NPV	TPR	TNR
**Serotype**	94.8%	-	-	-	-
IAB	98.5%	90.0%	99.2%	90.0%	99.2%
IC	97.0%	71.4%	100.0%	100.0%	96.8%
ID	95.6%	97.9%	94.3%	90.2%	98.8%
IE	98.5%	98.4%	98.6%	98.4%	98.6%
**Host type**	87.4%	-	-	-	-
large	87.4%	90.7%	85.9%	75.0%	95.2%
small	87.4%	85.9%	90.7%	95.2%	75.0%

(PPV) = TP/(TP + FP), (NPV) = TN/(TN+FN), TPR = TP/(TP+FN), and TNR = TN/(TN+FP).

The SNP at position 9201 corresponds to residue 213 on the E2 protein; substituting G for C at this locus was shown previously to mediate a shift from serotype ID to IC [11]. Although we also found that this locus provided the best discrimination between serotypes ID and IAB/IC, the association was not as clear as indicated by the previous studies. Three serotype ID genomes (R16905, 8138, and 204381) had an A at position 9201, corresponding to a lysine at residue 213, which in earlier studies was associated with serotypes IAB and IC.

The SNP at position 7764 lies within the capsid gene; an A or G at this position, corresponding to lysine or arginine at residue 68, is associated with serotypes IAB and IC respectively. The only strains not classified correctly by this SNP are the three serotype ID strains that are also misclassified by the SNP at 9201. The serotype data was obtained from previous studies, and it is possible that the serotypes were incorrectly determined.

To assess whether other loci would perform equally well for predicting serotype, we performed mutual information clustering to identify equivalence groups of loci, such that knowing the allele at one locus in a group completely determines the alleles of the other loci. A total of 4126 loci were present in the TC-83 genome and were polymorphic across all VEEV genomes. The largest equivalence group comprised 666 loci, which were those that have one allele for the serotype IE branch of the phylogenetic tree (including the 3 serotype ID outliers) and a different allele for the IAB/IC/ID branch. The remaining equivalence groups ranged in size from 2 to 76 loci; 2124 loci are singletons. The loci at positions 9987, 9201 and 7764 used in the serotype classifier are all singletons, having distinct patterns of alleles across the full set of isolates. The SNP at 9201 is also a singleton with respect to the 71 isolates in the non-IE subtree of the decision tree. However, across the 24 isolates in the IAB/IC subtree, there were 14 non-synonymous loci, 7 of which were in structural protein genes, which have the same allele pattern as 7764. Any of these loci would perform equally well in distinguishing serotype IAB and IC isolates, once the likely ID and IE isolates have been excluded by testing the loci at 9987 and 9201. Therefore, it would be premature to identify any one of these loci as determining the serotype IAB vs IC phenotype.

Host type prediction was less accurate than serotype prediction; 89.6% of strains were correctly predicted to have been collected from large mammals vs “small” hosts (mosquitos and rodents, including sentinel hamsters) (Table 3). This may reflect that hosts are sampled during outbreaks than during enzootic surveillance. The true positive rate (TPR) was larger for small hosts (95%) than for large hosts (81%). Close inspection of the SNP variants used in the decision tree classifier (Fig 3) showed that their allele patterns were associated with phylogenetic branches rather than host type, and no mutations that universally associated with host type across multiple different phylogenetic branches could be identified.

We also built classifiers in which the predictors were restricted to non-synonymous loci within the genes encoding structural proteins, or further restricted to envelope protein genes. Serotype classifiers based on structural protein loci were more accurate than envelope glycoprotein-restricted classifiers (data not shown), but not as accurate as unrestricted classifiers. For host type prediction, the best overall classifier used envelope protein loci only, so restricting to smaller locus sets had no effect.

### Comparison of single gene, MSA and SNP-based trees

We hypothesized that phylogenetic analyses of VEEV based on comparing single gene sequences, as was done in some earlier studies (2), would yield trees with lower resolution and differing topology than whole-genome MSA and SNP-based trees. To assess the impact of a single-gene approach, we compared the maximum parsimony tree based on all SNPs against trees generated using only the SNPs in each of the structural protein genes. We found that only 47% to 58% of the splits from the all-SNPs tree are present in any of the individual envelope gene trees (Table 4). Since only 9.5% to 14% of the SNPs occur within any of the envelope genes, the lower resolution of these trees is expected. The tree based on capsid gene SNPs had substantially worse resolution, however, with only 37% of the splits observed in the all-SNPs tree. Since the capsid gene contains over 3.5 times as many SNPs as the E3 gene, the number of splits shared by a gene-specific tree clearly depends on factors other than the total number of SNPs. The E1 gene resulted in the best representation of the tree, as it captures 58% of the splits identified in the all-SNPs tree.

**Table 4:** Comparison of trees from multiple sequence alignment versus all SNPs, and trees from SNPs located in a single gene versus all SNPs.

Tree comparison	Splits Found in 2^nd^ tree	Total Splits in SNP tree	Fraction splits in SNP tree found in 2^nd^ tree
All SNPs vs MSA	112	146	0.77
All SNPs vs E1	84	146	0.58
All SNPs vs E2	72	146	0.49
All SNPs vs E3	68	146	0.47
All SNPs vs capsid	54	146	0.37

To compare the topologies of trees generated with whole-genome SNPs or MSA to single-gene trees, we generated tanglegrams. S1 Fig shows a tanglegram with the MSA-based tree on the left and the all-SNPs tree on the right, with lines connecting the same taxa between trees. Differences between these trees were minor and within a reasonable expectation of uncertainty in the trees, mostly involving poorly resolved isolates such as Mucambo, CabassouCaAr, and PixunaBeAn. These isolates were collected from mosquito pools from 1954–1980 in French Guiana, Brazil, Argentina, and Peru, and are now considered different species in the VEE antigenic complex [26]. Each of these genomes has about 500 genome specific SNP alleles. They are the sole representatives of serotypes IF, IIIA, IIIB, IIIC, IV, V, and VI, each branching off the tree basal to the branches leading to the more heavily sequenced VEEV serotypes from Mexico, Peru, and Venezuela. In summary, the similarity between the whole genome SNP and MSA trees supports the SNP genotyping approach to phylogenetically characterize unsequenced samples using SNP arrays.

S2 and S3 Figs show tanglegrams with the all-SNPs tree on the left and the trees based on SNPs in the E1 (S2 Fig) or capsid (S3 Fig) gene on the right. The EEEV genomes were not clustered as a monophyletic group in any of the SNP gene trees, possibly because these genomes are too divergent from the VEEV genomes. Further, the capsid gene SNP tree had lower accuracy than the E1 gene tree, as indicated by the many crossing lines of the tanglegram in S3 Fig. The differences between the single gene and whole genome SNP trees illustrate the difficulty of phylogenetic analyses based on a small region rather than the full length of the genome, and suggest that SNP phylogenies based on single genes may have low resolution and accuracy.

### Microarray analysis of VEEV cDNA samples

To address the question of whether microarrays provide a viable alternative to whole-genome sequencing for VEEV strain characterization, we developed a VEEV SNP array. The array included 70,760 probes covering all 7,926 loci discovered with kSNP. We hybridized cDNAs from 134 isolates to SNP arrays. Genome sequences were available for 81 of the isolates. We calculated overall concordance rates between the allele calls made by SNP microarray versus those called by whole genome sequences; these are summarized in S3 Table. The overall concordance rate was 96.2%. Hybridizations of replicate cDNA samples extracted from three isolates showed close agreement between replicates. The array correctly classified 76 out of 84 cDNA samples. Four of the 8 misclassified cases were highly similar sequences collected in the same location. One source of error was that the array analysis currently is not able to call a locus as missing, even if that locus is not present in the genome sequence, causing discordance between the genome and array genotypes.

A potential advantage of microarray analysis over DNA sequencing is its reduced need for viral isolation and culturing, allowing viruses to be characterized directly from a tissue sample. To test whether this was feasible, we isolated RNA from the brains of 3 replicate mice infected with VEEV strain TC-83, analyzed the RNA using the SNP microarray, and compared the array genotypes to our panel of 144 sequence-based genotypes. For all 3 replicates, the array genotypes were closest to those of the published sequence for the TC-83 strain, as shown in S4 Table. This suggests that a SNP microarray can produce accurate VEEV genotypes, even in the presence of a complex host DNA background.

## Discussion

Tools for rapid genotyping of equine encephalitis virus strains and elucidating their phylogenetic relationships are critically important to understand why certain strains are likely to cause epizootic infection, and to forecast the incidence of potential epidemic events. The results above represent analyses of VEE complex strains derived from a wide range of hosts and geographic regions. The collected data indicate that our microarray and sequencing-based genotyping tools effectively distinguish VEEV strains and allow us to cluster those strains according to their derivation and phenotypic history.

Since the VEEV genome is small, whole genome multiple sequence alignment (MSA) of more than 140 sequences was feasible. Predicted genotype/phenotype associations were slightly more accurate when genotypes were based on variable positions in a whole genome alignment than when they were based on *k*-mer contexts defined by kSNP (data not shown). The MSA approach is usually not feasible for bacterial genomes, so that kSNP is typically a better option for bacterial genotype/phenotype association studies.

Relying on non-random associations between serotype and sequence variation, we were able to build decision trees to predict VEEV serotype. With 3 loci, prediction accuracy was 95.6% to 98.5%. However, strains that clustered phylogenetically with a different serotype were sometimes mislabeled by the decision trees. In addition, there were multiple loci that distinguished equally well between IAB and IC strains, after excluding isolates with a T at position 9987 (which are mostly serotype IE) or a C at position 9201 (which are mostly ID). These observations suggest that the actual amino acid variants that determine serotype may be any of a wide range of candidates, as suggested earlier [12], and that the association we observe between serotype and certain other variants is due to their co-inheritance with the causal variants. We also noted that no variants associated perfectly with any of the serotypes; thus it must be possible to obtain the same shift in antigenic specificity from mutations at multiple loci.

A previous study [11] based on a smaller set of VEEV genomes investigated the mutations required for the virus to transition from the enzootic cycle (small mammals, *Culex* mosquitos, forest habitats) to the epizootic cycle (*Aedes/Psorophora* mosquitos, amplification in equids, transmission to humans). It reported that a single mutation in the E2 protein (T213 -> K or R), when engineered into a serotype ID enzootic strain, changed its serotype to IC and rendered it capable of causing enhanced viremia in horses, as well as possibly more efficiently infecting *Aedes* (*Ochlerotatus*) mosquitos implicated as vectors in equine-amplified epizootics. Our results, based on the larger set of VEEV genomes available now, suggest a more complex association between genotype and phenotype. We identified three serotype ID strains with the K213 E2 allele. There was no single locus that distinguished all of these ID strains from the IAB and IC strains nearest to them phylogenetically. This is further evidence that a variety of mutations can mediate the shift from ID to IAB or IC serotypes.

Comparison of the phylogenetic tree predicted from whole genome SNPs was similar to that from whole genome multiple sequence alignment. Narrowing to single gene SNP trees showed that the E1 gene SNPs more closely represent the whole genome SNP tree than do the SNPs from the other envelope protein or capsid genes. This concurs with previous analyses based on sequence alignment rather than SNPs, which also showed that the E1 gene captures the same high level relationships as the whole genome alignment but does not provide the same resolution [28]. However, these results emphasize that use of a small region of the genome for SNP analysis provides lower resolution than whole genome SNPs, and with some genes even results in different tree topology. A whole genome SNP approach more effectively represents complete phylogenetic relationships to reveal distinctions that would otherwise be overlooked.

A *k*-mer based approach to SNP discovery has limitations relative to full sequence alignment, particularly for highly variable RNA viruses. However, our comparison of data derived from multiple sequence alignments versus SNP analysis revealed that the resultant trees were very similar and reliably identified comparable splits. These observed similarities are important in that they support the use of our unique SNP array as an effective detection and genotyping tool without available whole genome sequence data. The SNP array results can be obtained within 24 hours as compared to 48–72 hours by whole genome sequencing. The cost of running a sample on SNP array is roughly 10 times less than whole genome sequencing. We have shown here that data obtained from SNP arrays are capable of reliably clustering strains in accordance with their respective whole genome sequence data. Array data provide sufficient accuracy in phylogenetic classification to correctly cluster isolates by clade and to identify the closest neighbors that have been sequenced or hybridized to the array. This technology would be particularly useful for rapidly evaluating a novel strain from an epizootic outbreak event. Further evaluation of the SNP array, using unknown or unsequenced VEEV strains, could provide additional validity and value of this technology in detection and genotyping of outbreak strains.

## Supporting Information

S1 Fig Tanglegram connecting the corresponding taxa which illustrates the high similarity between the MSA tree (left) and the SNP tree (right). (DOCX)

S2 Fig Tanglegram illustrating where the SNP tree based on all the SNPs (left) and that based only on the SNPs in the E1 gene (right) differ. (DOCX)

S3 Fig Tanglegram illustrating where the SNP tree based on all the SNPs (left) and that based only on the SNPs in the capsid gene (right) differ. (DOCX)

S1 Table Characteristics of VEE antigenic complex strains used for whole genome SNP analysis and/or tested on SNP microarray. (XLSX)

S2 Table Annotations, 13-mer contexts and reference genome alignments for SNPs identified by whole genome analysis. (XLSX)

S3 Table Concordance of array and genome-based allele calls, for non-passaged isolates with known genome sequences. Rows in bold text indicate replicate arrays for the same isolates.
(XLSX)

S4 Table Comparison of genotypes for VEEV on tissue from TC-83 infected mice. (XLSX)

## References

[1] Weaver SC, Ferro C, Barrera R, Boshell J, Navarro JC. Venezuelan equine encephalitis. Annu Rev Entomol. 2004;49:141–74. . 14651460 10.1146/annurev.ento.49.061802.123422

[2] Quiroz E, Aguilar PV, Cisneros J, Tesh RB, Weaver SC. Venezuelan equine encephalitis in Panama: fatal endemic disease and genetic diversity of etiologic viral strains. PLoS Negl Trop Dis. 2009;3(6):e472 10.1371/journal.pntd.0000472 19564908 PMC2697379

[3] Vilcarromero S, Aguilar PV, Halsey ES, Laguna-Torres VA, Razuri H, Perez J, et al Venezuelan equine encephalitis and 2 human deaths. Peru Emerg Infect Dis 2010;16:553–6. 10.3201/eid1603.090970 20202445 PMC3322018

[4] Johnson KM, Shelokov A, Peralta PH, Dammin GJ, Young NA. Recovery of Venezuelan equine encephalomyelitis virus in Panama. A fatal case in man. The American journal of tropical medicine and hygiene. 1968;17(3):432–40. . 5690051 10.4269/ajtmh.1968.17.432

[5] Ferro C, Olano VA, Ahumada M, Weaver S. [Mosquitos (Diptera: Culicidae) in the small village where a human case of Venezuelan equine encephalitis was recorded]. Biomedica. 2008;28(2):234–44. Epub 2008/08/23. doi: S0120-41572008000200008 [pii]. . 18719725

[6] Aguilar PV, Greene IP, Coffey LL, Medina G, Moncayo AC, Anishchenko M, et al Endemic Venezuelan Equine Encephalitis in Northern Peru. Emerging Infectious Diseases. 2004;10(5):880–8. 10.3201/eid1005.030634 . 15200823 PMC3323213

[7] Forshey BM, Guevara C, Laguna-Torres VA, Cespedes M, Vargas J, Gianella A, et al Arboviral Etiologies of Acute Febrile Illnesses in Western South America, 2000–2007. PLoS Negl Trop Dis. 2010;4(8):e787 10.1371/journal.pntd.0000787 20706628 PMC2919378

[8] Vilcarromero S, Laguna-Torres A, Fernández C, Gotuzzo E, Suárez L, Céspedes M, et al Venezuelan Equine Encephalitis and Upper Gastrointestinal Bleeding in Child. Emerging Infectious Diseases. 2009;15(2):323–5. 10.3201/eid1502.081018 . 19193285 PMC2657634

[9] Watts DM, Lavera V, Callahan J, Rossi C, Oberste MS, Roehrig JT, et al Venezuelan equine encephalitis and Oropouche virus infections among Peruvian army troops in the Amazon region of Peru. Am J Trop Med Hyg. 1997;56:661–7. 9230800 10.4269/ajtmh.1997.56.661

[10] Watts DM, Lavera V, Callahan J, Rossi C, Oberste MS, Roehrig JT, et al Venezuelan equine encephalitis febrile cases among humans in the Peruvian Amazon River region. Am J Trop Med Hyg 1998;58:35–40. 9452289 10.4269/ajtmh.1998.58.35

[11] Anishchenko M, Bowen RA, Paessler S, Austgen L, Greene IP, Weaver SC. Venezuelan encephalitis emergence mediated by a phylogenetically predicted viral mutation. Proceedings of the National Academy of Sciences of the United States of America. 2006;103(13):4994–9. 10.1073/pnas.0509961103 16549790 PMC1458783

[12] Brault AC, Powers AM, Holmes EC, Woelk CH, Weaver SC. Positively Charged Amino Acid Substitutions in the E2 Envelope Glycoprotein Are Associated with the Emergence of Venezuelan Equine Encephalitis Virus. Journal of Virology. 2002;76(4):1718–30. 10.1128/JVI.76.4.1718-1730.2002 . 11799167 PMC135911

[13] Gardner S, Slezak T. Scalable SNP Analyses of 100+ Bacterial or Viral Genomes. J Forensic Res. 2010;1:107, 10.4172/2157-7145.1000107

[14] Gardner SN, Hall BG. When Whole-Genome Alignments Just Won't Work: kSNP v2 Software for Alignment-Free SNP Discovery and Phylogenetics of Hundreds of Microbial Genomes. PLoS ONE. 2013;8(12):e81760 10.1371/journal.pone.0081760 24349125 PMC3857212

[15] Edgar RC. MUSCLE: multiple sequence alignment with high accuracy and high throughput. Nucleic Acids Res. 2004;32:1792–7. 15034147 10.1093/nar/gkh340 PMC390337

[16] Price MN. Fast Tree-Comparison Tools Berkeley, CA. Available: http://meta.microbesonline.org/fasttree/treecmp.html.

[17] Huson DH, Scornavacca C. Dendroscope 3: an interactive tool for rooted phylogenetic trees and networks. Syst Biol. 2012;61(6):1061–7. 10.1093/sysbio/sys062 . 22780991

[18] Venkatachalam B, Apple J, St John K, Gusfield D. Untangling tanglegrams: comparing trees by their drawings. IEEE/ACM Trans Comput Biol Bioinform. 2010;7:588–97. 10.1109/TCBB.2010.57 20530818

[19] Gardner SN, Thissen J, McLoughlin K, Slezak T, Jaing C. Optimizing SNP microarray probe design for high accuracy microbial genotyping. J Microbio Meth. 2013:10.1016/j.mimet.2013.07.006 23871857

[20] Jaing C, Gardner SN, McLoughlin K, Mulakken N, Alegria-Hartman M, Banda P, et al A functional gene array for detection of bacterial virulence elements. PLoS ONE. 2008;3(5):e2163 10.1371/journal.pone.0002163 18478124 PMC2367441

[21] Berge TO, Banks IS, Tigertt WD. Attenuation of Venezuelan equine encephalomyelitis virus by in vitro cultivation in guinea pig heart cells. Am J Hyg. 1961;73:209–18.

[22] Team RC. R: A language and environment for statistical computing R Foundation for Statistical Computing Vienna, Austria 2014 Available: http://www.R-project.org/.

[23] Therneau T, Atkinson B, Ripley B, Oksanen J, De'ath G. mvpart: Multivariate partitioning. R package version 1.6–1 2013. Available: http://cran.R-project.org/package=mvpart.

[24] Venables W, Ripley B. Modern Applied Statistics with S. 4 ed. New York: Springer; 2002.

[25] Kolaczkowski B, Thornton JW. Performance of maximum parsimony and likelihood phylogenetics when evolution is heterogeneous. Nature. 2004;431:980–4. 15496922 10.1038/nature02917

[26] Weaver SC, Barrett AD. Transmission cycles, host range, evolution and emergence of arboviral disease. Nature reviews Microbiology. 2004;2(10):789–801. 10.1038/nrmicro1006 . 15378043 PMC7097645

[27] Weaver S, Pfeffer M, Marriott K, Kang W, Kinney R. Genetic evidence for the origins of Venezuelan equine encephalitis virus subtype IAB outbreaks. Am J Trop Med Hyg. 1999;60(3):441–8. 10466974 10.4269/ajtmh.1999.60.441

[28] Wolfe DN, Heppner DG, Gardner SN, Jaing C, Dupuy LC, Schmaljohn CS, et al Current Strategic Thinking for the Development of a Trivalent Alphavirus Vaccine for Human Use. The American Journal of Tropical Medicine and Hygiene. 2014 10.4269/ajtmh.14-0055 PMC4155543 24842880

