# Supplementary figures and images for: Characterization of Genetic Variability of Venezuelan Equine Encephalitis Viruses
Source: PLoS One. 2016 Apr 7;11(4):e0152604. doi: 10.1371/journal.pone.0152604 (PMC4824352; doi:10.1371/journal.pone.0152604)

Figure S1


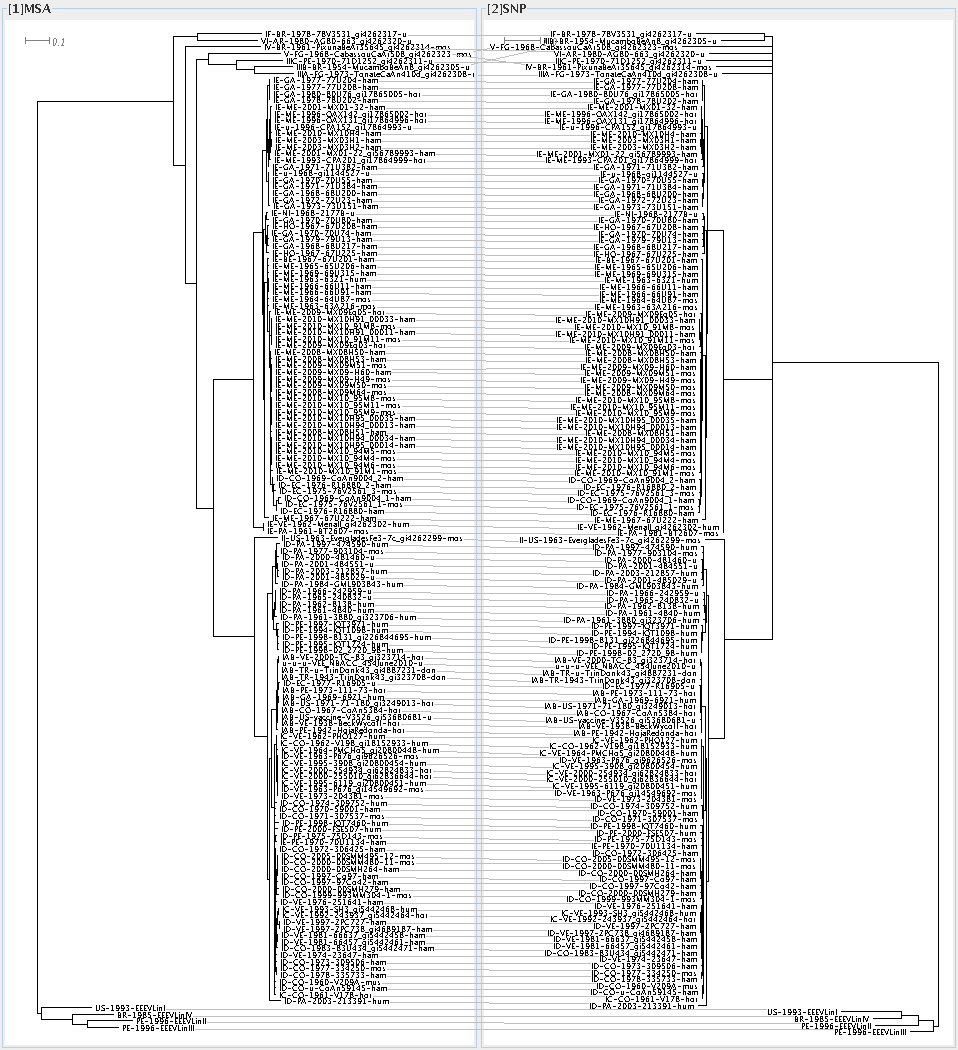

Supplement: S1 Fig — (DOCX) [file pone.0152604.s001.docx]

Figure S2


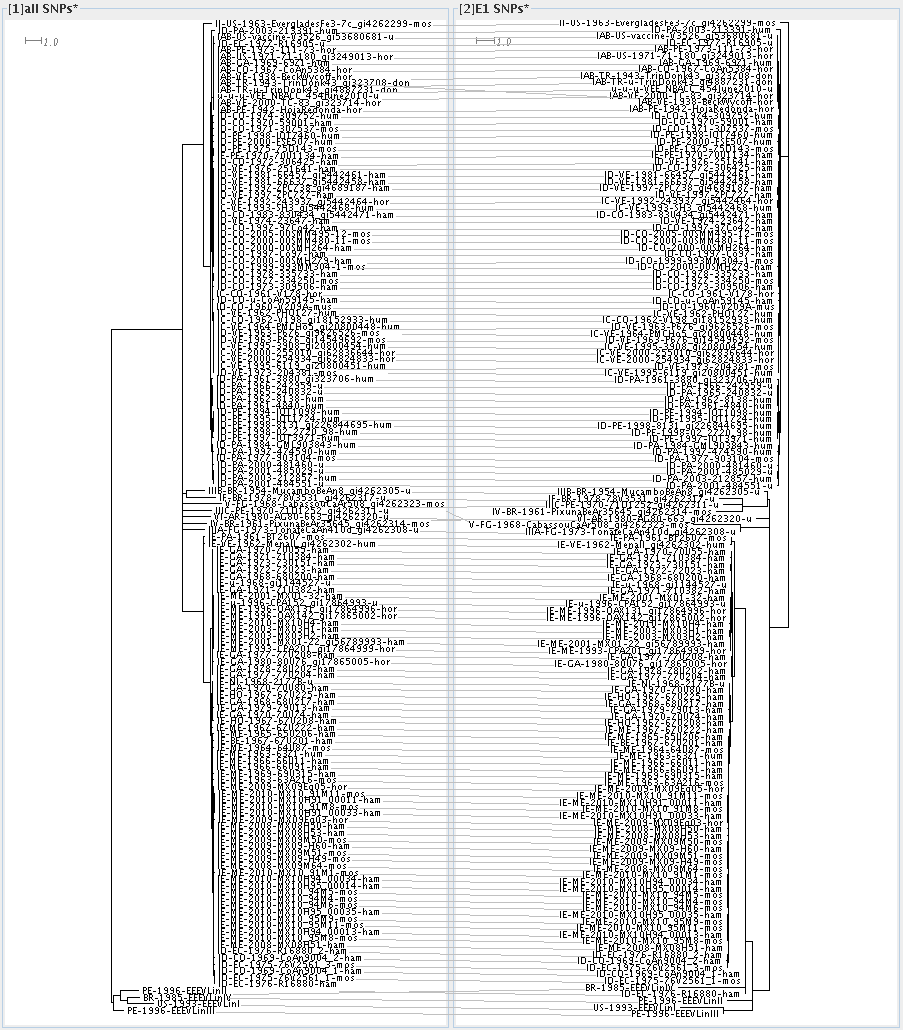

Supplement: S2 Fig — (DOCX) [file pone.0152604.s002.docx]

Figure S3


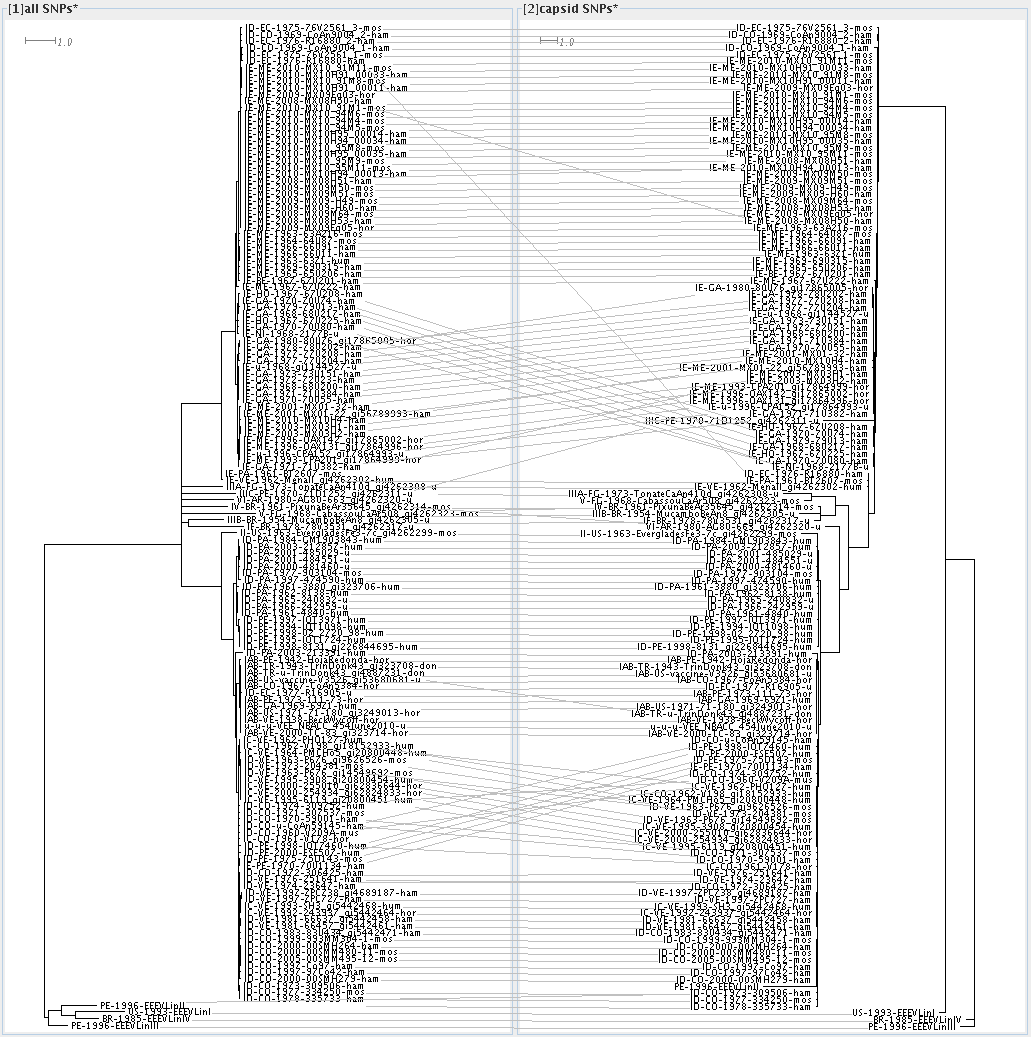

Supplement: S3 Fig — (DOCX) [file pone.0152604.s003.docx]
